# Leveraging Machine Learning for Advanced Nanoscale X-ray Analysis: Unmixing Multicomponent Signals and Enhancing Chemical Quantification

**DOI:** 10.1021/acs.nanolett.4c02446

**Published:** 2024-08-06

**Authors:** Hui Chen, Duncan T. L. Alexander, Cécile Hébert

**Affiliations:** Electron Spectrometry and Microscopy Laboratory (LSME), Institute of Physics (IPHYS), École Polytechnique Fédérale de Lausanne (EPFL), 1015 Lausanne, Switzerland

**Keywords:** STEM, EDX, NMF, pan-sharpening, minerals, nanocatalysts

## Abstract

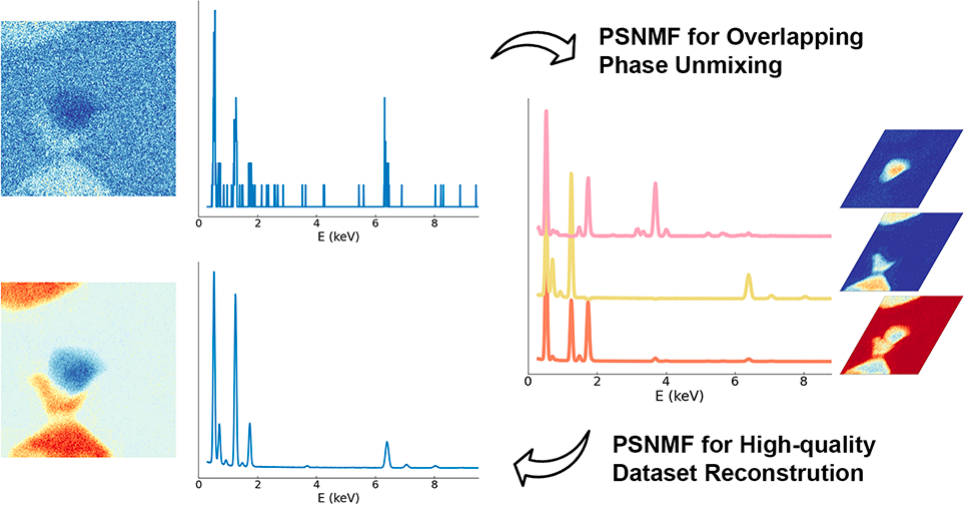

Energy dispersive X-ray (EDX) spectroscopy in the transmission electron microscope is a key tool for nanomaterials analysis, providing a direct link between spatial and chemical information. However, using it for precisely determining chemical compositions presents challenges of noisy data from low X-ray yields and mixed signals from phases that overlap along the electron beam trajectory. Here, we introduce a novel method, non-negative matrix factorization based pan-sharpening (PSNMF), to address these limitations. Leveraging the Poisson nature of EDX spectral noise and binning operations, PSNMF retrieves high-quality phase spectral and spatial signatures via consecutive factorizations. After validating PSNMF with synthetic data sets of different noise levels, we illustrate its effectiveness on two distinct experimental cases: a nanomineralogical lamella, and supported catalytic nanoparticles. Not only does PSNMF obtain accurate phase signatures, but data sets reconstructed from the outputs have demonstrably lower noise and better fidelity than from the benchmark denoising method of principle component analysis.

An energy-dispersive X-ray spectrum (EDX spectrum) is obtained by bombarding a sample with a beam of high-energy electrons that induces the emission of characteristic X-rays from the sample’s atoms.^1^ These emitted X-rays are then measured and analyzed, both to identify the elements present in the material and to quantify their relative abundance. By combining EDX with scanning transmission electron microscopy (STEM-EDX), a sub-nm or even atomic spatial resolution image can be obtained for element mapping.^2^ As a result, this technique is widely employed for the chemical characterization of nanomaterials, playing an essential role in understanding their properties and performance by determining the elemental compositions of their constituent phases or compounds.^3−7^ Nevertheless, the technique poses challenges. When quantifying nanomaterials with STEM-EDX, the signal-to-noise ratio (SNR) of the spectral signal is often limited due to two main factors: first, the very small sample volume interacting with the focused electron beam; second, limitations on the electron dose to avoid beam-induced damage. Moreover, it is difficult to quantify heterogeneous nanostructures or nanocomposites using STEM-EDX when different phases overlap along the trajectory of the electron beam through the sample, resulting in EDX signals that derive from a mixture of phases. For precise compositional determination of each phase, it is vital to separate these mixed signals.

To address these challenges, machine learning algorithms are increasingly used in the analysis of STEM-EDX mapping data. Among these, principal component analysis (PCA)^8^ has arguably become the *de facto* “gold standard” for denoising data sets.^9−11^ Nevertheless, it is prone to introducing artifacts into reconstructed data resulting from its inability to ensure signal positivity.^12,13^ Independent component analysis (ICA)^14^ and non-negative matrix factorization (NMF)^15^ are in turn commonly utilized for identifying components from mixed or overlapped phases.^16−19^ However, these methods also have limitations: data noise can lead to incorrect outputs;^20,21^ and, if the chemical signals from individual phases are too similar, the capacity of ICA and NMF to disentangle them can prove poor.^22−24^

Pan-sharpening (PS) is a data-fusion technique widely used in satellite imaging,^25^ which combines the high spatial resolution of a panchromatic image with the color information from a multispectral image to produce a single, high-resolution, color-enhanced image. Recently, Borodinov et al. adapted this concept for the enhanced analysis of STEM electron energy-loss spectroscopy (EELS) plasmonic data sets.^26^ Similarly to the original PS methodology, their implementation depends on acquiring two data sets, one having high spectral fidelity but few spatial pixels, and the other having high spatial resolution but poor spectral signal. Now considering STEM-EDX, one specificity of the data sets is that the spectral noise has a Poissonian nature.^27,28^ A data set having high spatial resolution but poor spectral signal can therefore be transformed into one having high spectral fidelity but fewer spatial pixels by simple spatial binning, all while keeping a Poissonian noise structure. Our new method, which we call PSNMF (NMF-based pan-sharpening), combines such binning operations with PS-inspired data fusion to leverage the strengths of NMF, notably its non-negativity and component separation capabilities. With this, we achieve both a highly effective unmixing of signals from overlapped phases and a very efficient signal denoising.

The exact workflow is presented in Figure 1. We refer to the original data set as HR-LS (high-resolution, low-signal). In this data set, the EDX spectrum of each single pixel has a very low SNR given that, typically, it has just one to a few hundred X-ray counts when summed over its whole spectral energy range. From this HR-LS data set, we perform *b* × *b* pixel spatial binning to generate a new data set. This binning, while reducing spatial resolution, improves the SNR in each pixel’s spectrum. It does so by increasing the signal in each spectrum by a factor of *b*^2^. Since Poisson noise is proportional to the square root of the counts, the SNR per pixel is therefore improved by a factor of *b*. The binned data set is referred to as LR-HS (low-resolution, high-signal). By strategically fusing the algorithmic decomposition outputs from the complementary data sets, HR-LS and LR-HS, we will reconstruct a high-quality data set, denoted as HR-HS (high-resolution, high-signal). While PS fusion can be implemented through several methods, here we combine it with NMF, due to its ability to unmix signals while ensuring a physically correct non-negativity that helps produce meaningful outputs. In all, the PSNMF procedure comprises four steps. These steps are now described, while a mathematical description of them is given in Supporting Information, Note 1.

1. A spatial binning of the original HR-LS data set by a factor *b* is applied to create a LR-HS data set. *b* can be large, since it does not affect the spatial resolution of the final reconstructed data set. As a rule of thumb, the maximum *b* is that which preserves individual spatial phase structures as distinctly as possible, without introducing additional mixing through binning. It is advisible to test several values of *b*, up to this maximum.
2. A first NMF decomposition is applied to the binned LR-HS data set, producing spectral components with significantly improved accuracy when compared to an NMF decomposition performed on the original, noisier HR-LS data set.
3. A second NMF decomposition is executed on the original HR-LS data set to recover high spatial resolution abundance maps for the components. Critically, the spectral components derived from the first NMF decomposition are used to initialize the algorithm. Given that NMF operates as a heuristic algorithm and is, for noisy data sets, highly sensitive to initial values, this greatly improves the decomposition result when compared to that from a random initialization on the same data set.
4. Finally, we fuse the high-accuracy spectral components delivered by the first NMF decomposition with the high spatial resolution abundance maps obtained from the second NMF decomposition. This produces a high quality reconstructed data set, having a greatly enhanced SNR for the EDX signal while retaining the full spatial resolution of the original data set.

**Figure 1:**
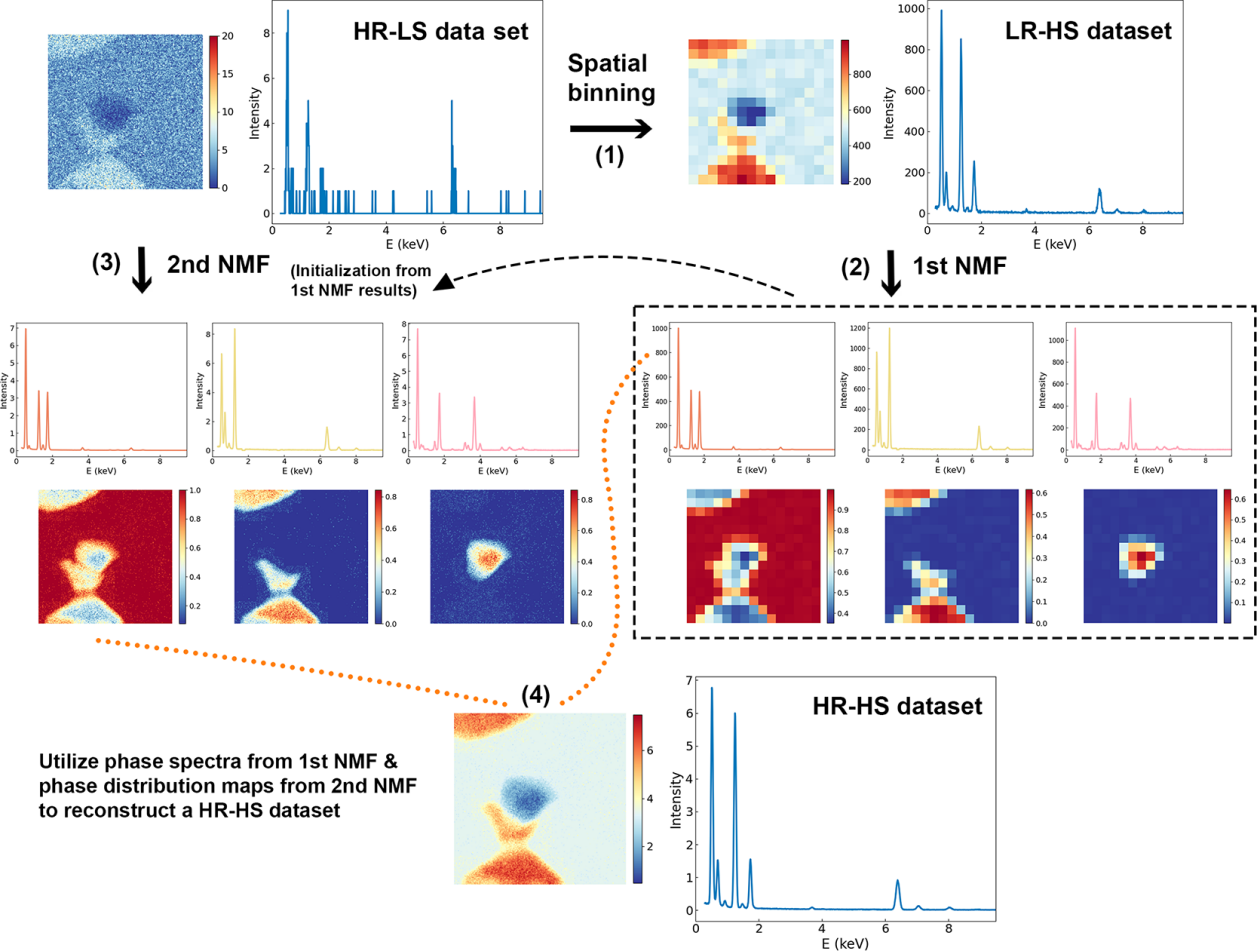
Schematic illustration of PSNMF methodology. From an initial STEM-EDX data set (HR-LS), binning using spatial factor *b* is applied to create an LR-HS data set with an improved SNR for the pixel spectra. Performing a first NMF on the LR-HS data set generates spatial and spectral components, of which the spectral components are high-quality. These are used to initialize a second NMF decomposition of the HR-LS data set, delivering low noise, high-resolution spatial components (maps). A final, high-quality reconstructed data set is made by combining the spectra of the first NMF with the spatial distribution maps of the second NMF.

We first illustrate and quantitatively test the capabilities of PSNMF by applying it to synthetic data sets having a known ground truth. Our choice of test case for constructing the synthetic data set was based on the following considerations. First, we wanted a case that represents usual challenges encountered in the chemical analysis of samples obtained by solidification processes, as are typical for many areas of geology and materials science. Second, we selected a sample type that we have extensively studied, such that, with high confidence, we can produce a very realistic synthetic data set. We therefore simulate a STEM-EDX data set that mimics those acquired from mineral assemblages that are solidified under high pressure and temperature conditions for the study of Earth’s deep mantle.^29^ It comprises three distinct mineral phases that form and segregate with nanoscale spatial distributions: bridgmanite, ferropericlase, and calcium perovskite.^30^ Bridgmanite (Brg), a silicate perovskite often denoted as (Mg,Fe)SiO_3_, stands as the predominant mantle mineral. Notably, it may incorporate minor quantities of Al and Ca and also trace quantities of rare-earth elements such as Nd and Sm.^31^ Ferropericlase (Fp), chemically expressed as (Mg,Fe)O, constitutes the second most abundant mineral in the lower mantle. The mineral with the smallest volume fraction is calcium perovskite (CaPv). It has a chemical formula of CaSiO_3_, and acts as the primary host for rare earth elements within the mantle. The analysis of these mineral phases greatly enhances our understanding of Earth’s geochemical evolution.^32,33^

Table 1 presents the exact compositions set for Brg, Fp, and CaPv for creating the synthetic data set. Figure 2 shows their designed spatial distributions (“Ground truth” column), which are based on those previously identified from the in-depth investigation of an experimental sample.^34^ As shown in the figure, there are many regions in which different phases overlap spatially. Together with the compositional overlaps listed in Table 1, this creates a basis that is typically challenging for effective data decomposition into correct phase signatures. From the phase compositions and distributions, a noise-free STEM-EDX data set of 180 × 180 pixels size is simulated using the “espm” (Electron Spectro-Microscopy) open-source Python library.^35^ Using the same software package, Poisson sampling is then applied to generate two data sets with realistic spectral noise. One data set exhibits a moderate SNR—corresponding to what can typically be achieved experimentally on such samples—while the other has an extremely low SNR. In the medium SNR data set, the average X-ray count per pixel is 147. In contrast, the extremely low SNR data set has an average of 18 X-ray counts per pixel. The raw elemental maps derived from these two synthetic data sets are provided in the Supporting Information Figures S1 and S2. Before applying PSNMF to the synthetic data sets, we conducted PCA to determine the appropriate number of components for the NMF decomposition. In agreement with the three phases used to generate the data sets, the scree plots, included in the Supporting Information Figure S3, indicate the presence of three components, albeit with varying variances.

**Figure 2:**
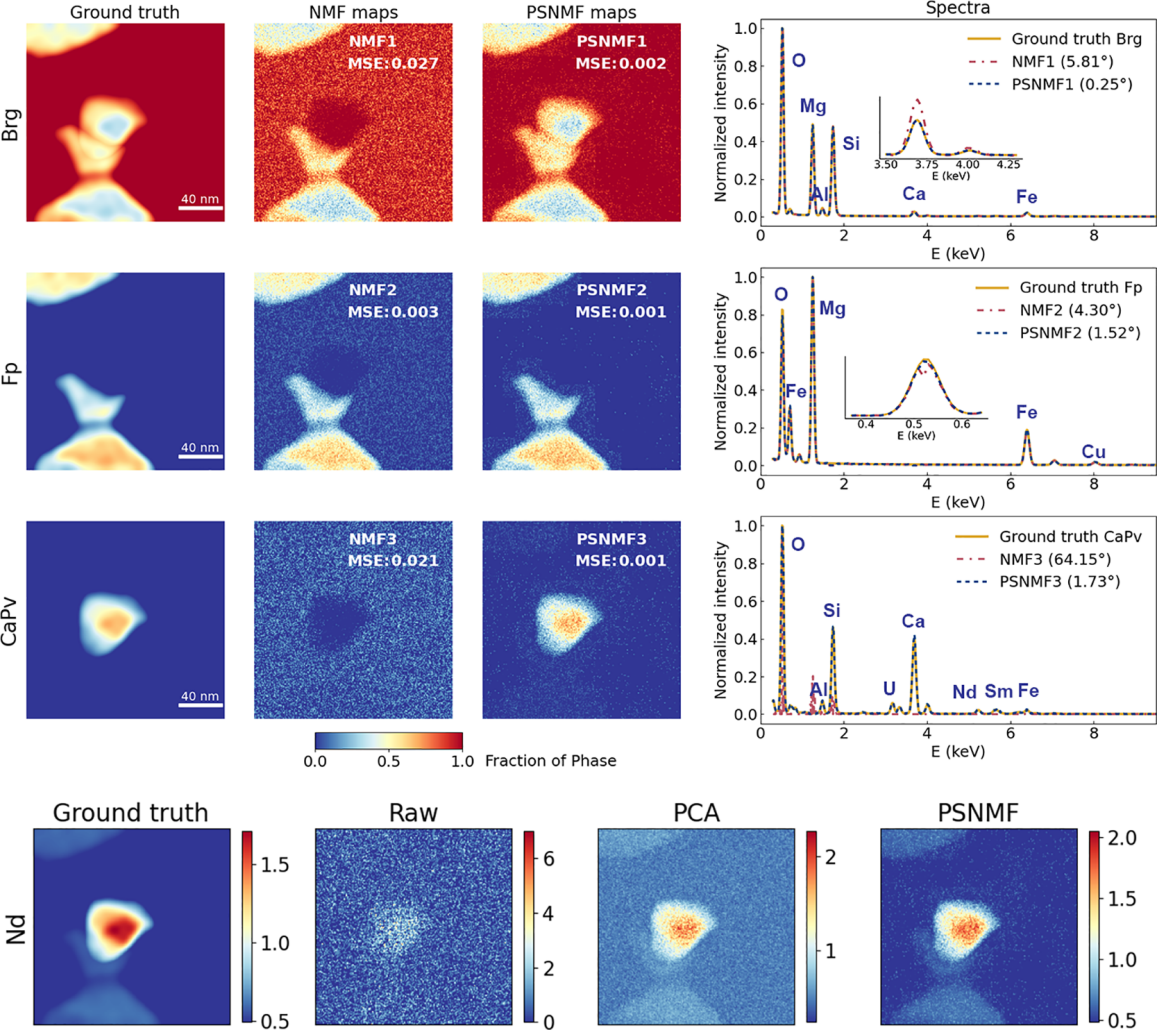
Application of PSNMF to a simulated data set having medium SNR (average of 147 X-ray counts per pixel), respectively compared to standard NMF for phase identification and PCA for data set denoising. The PSNMF uses *b* = 12. On the upper left, abundance maps of components from NMF and PSNMF are compared to ground truth phase maps, with phase spectral comparisons shown on the right. Note that the spectral legends and spatial loading maps display their respective spectral angles and MSE values. The two insets display magnified views of the Ca peaks in the Brg phase and the O peak in the Fp phase. On the bottom row, raw, PCA-denoised and PSNMF-denoised Nd maps are compared to the ground truth map.

**Table 1:** Compositions of the Three Simulated Phases Utilized in Generating the Synthetic Data Sets and the Compositions of PSNMF Components When Applied to the Two Synthetic Data Sets, Using *b* = 12 for the Medium SNR Data Set and *b* = 15 for the Low SNR Data Set

	Atomic %	Mg	Si	Al	Ca	Fe	Nd	Sm	U	O	Cu
	Brg	18.50	18.20	1.50	1.00	1.00	0.10	0.10	-	60.00	-
Phase	Fp	39.00	-	-	-	10.00	-	-	-	50.00	1.00
	CaPv	-	17.50	2.50	16.20	1.00	1.00	1.00	1.00	60.00	-
	PSNMF1	18.49	18.10	1.47	1.01	0.99	0.10	0.10	-	59.72	-
Medium SNR	PSNMF2	39.53	-	-	-	10.46	-	-	-	48.84	1.16
	PSNMF3	-	17.42	2.20	16.57	0.98	0.99	0.96	0.99	59.81	-
	PSNMF1	18.47	18.29	1.43	1.01	0.99	0.10	0.12	-	59.55	-
Low SNR	PSNMF2	39.65	-	-	-	10.36	-	-	-	48.63	1.26
	PSNMF3	-	17.43	2.41	15.47	1.05	0.99	0.91	1.08	60.25	-

First, we employ PSNMF on the data set with medium SNR, with incremental binning factors (*b* = 2, 4, 12, 15, 30). Upon reaching *b* = 12, PSNMF demonstrates an effective phase unmixing. This can be seen visually in Figure 2, where the resulting spectral components (PSNMF#) are compared to the spectra of the ground truth phases. As comparison, we also plot the results of a standard NMF decomposition performed on the same data set. Qualitatively, the PSNMF spectral match is substantially improved compared to that of NMF, particularly for the phase with the lowest volume fraction (CaPv) where the NMF3 spectrum shows a large deviation from the ground truth. To assess this quantitatively, we use a spectral angle metric,^36^ defined as
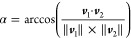
1where ***v***_1_ and ***v***_2_ are two spectral vectors of the same dimension. A smaller angle indicates a higher degree of spectral similarity; an angle of 0° indicates an exact match, while an angle of 90° implies no similarity. The close match of PSNMF1, PSNMF2, and PSNMF3 to the ground truths is confirmed by their respective spectral angles of 0.25°, 1.52°, and 1.73°. In comparison, as shown in Figure 2, the spectral angles for standard NMF are far greater, with values of 5.81°, 4.30°, and 64.15°, respectively. The last value in particular confirms that standard NMF fails to correctly extract the minor phase CaPv. It is also evident that NMF overestimates Ca in Brg and underestimates O in Fp, as shown in the insets of the spectra in Figure 2. This discrepancy does not occur for the components retrieved with PSNMF. Additionally, we quantify the three PSNMF components and list their compositions in Table 1. A comparison to the ground truth compositions shows that, impressively, even trace elements within Brg, such as Nd and Sm, are accurately quantified. These elemental quantifications were performed using the Cliff-Lorimer ratio method,^37^ employing k-factors derived from X-ray emission cross sections that were generated through state-of-the-art calculations using the “emtables” (Electron Microscopy Tables) library.^35^ One should note that the k-factors are the same as those used for constructing the synthetic data sets, hence discrepancies between quantification after reconstruction of a noisy data set can only be explained by a failed matrix decomposition.

Under the PSNMF methodology, the second NMF decomposition yields the high-spatial-resolution components, as shown in the PSNMF maps in Figure 2. Similarly to the spectra, these are compared to the abundance maps from a standard NMF decomposition. It is immediately seen that PSNMF retrieves phase distributions with much higher fidelity and lower noise than the standard NMF. This is particularly true for CaPv, where standard NMF largely fails to retrieve the phase distribution. To compare outputs quantitatively, we use a mean squared error (MSE) metric^36^ that measures the accuracy of the retrieved components compared to ground truth phase distribution maps. MSE is defined as
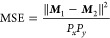
2where ***M***_1_ and ***M***_2_ are two maps of dimension *P*_*x*_ × *P*_*y*_ respectively representing the retrieved and ground truth abundance maps of a phase. The MSE can take values between 0 and 1, where 0 represents a perfect agreement between the two maps. The MSE values reported in Figure 2 confirm the remarkable ability of PSNMF to correctly retrieve the phase distributions. This is not the case for standard NMF, whose MSE values are an order of magnitude greater for the Brg and CaPv phases.

Having studied the power of PSNMF for phase unmixing, we now consider its use for data set denoising. The spectral and spatial components generated by PSNMF are used to reconstruct a high-quality STEM-EDX data set, from which elemental maps are extracted. As comparison, we apply PCA to the original data set, since this remains the “gold standard” tool for hyperspectral data denoising. The data set is then reconstructed using the first three components of the PCA decomposition. From the raw and reconstructed data sets, Figure 2 compares the resulting maps for a trace element, Nd. In the raw Nd map, because of the low spectral signal, noise is prominent, and the true spatial distribution cannot be distinguished. While PCA enhances the Nd map by decreasing noise and revealing the basic Nd distribution, the PSNMF-denoised Nd map clearly exhibits the least noise and closest fidelity to the ground truth map. The comparison of all elemental maps in the Supporting Information Figures S4 and S5 confirms the superior denoising by PSNMF, across the sample’s full elemental range.

We now extend our evaluation of the PSNMF methodology by testing it on the second synthetic data set having a very low SNR. The results are summarized and presented in Figure 3. As before, we compare outputs to those of standard NMF and PCA. From visual inspection of the results, and the spectral angles of 29.69° to 75.41°, it is evident that, because of the high noise level, NMF fails to correctly retrieve any of the phases. However, by applying *b* = 15, PSNMF yields phase unmixing results that are highly satisfactory given the poor quality of the initial data set, with spectral angles ranging from just 0.64° up to 5.04°. Remarkably, as presented in Table 1, quantification of the three PSNMF component spectra yields compositions that show minimal deviation from the designed composition. Therefore, we find that PSNMF is extremely effective for unmixing spatially overlapping and chemically similar phases even in the presence of high noise levels. As before, we use the three PSNMF components to reconstruct the STEM-EDX data set. From this, Figure 3 shows the extracted Nd elemental map, compared to ground truth, raw and PCA-denoised maps. While the raw Nd map exhibits only noise, the map from the PSNMF-reconstructed data set once again retrieves the true Nd distribution, with superior denoising compared to the map generated by PCA. A full comparison of ground truth, raw, and PCA- and PSNMF-denoised elemental maps can be found in Supporting Information Figures S6 and S7.

**Figure 3:**
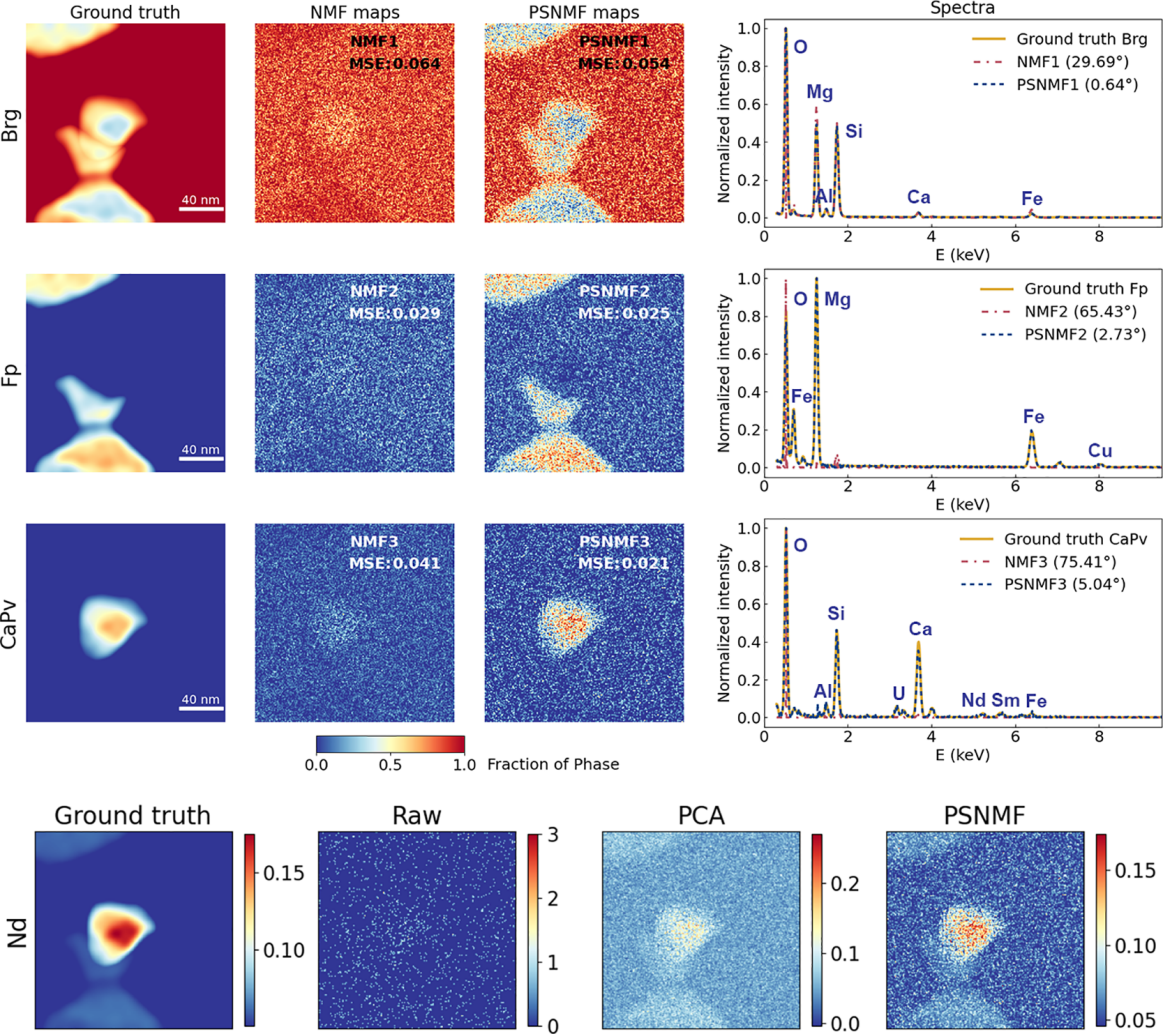
PSNMF applied to a simulated data set having a very low SNR (average of 18 X-ray counts per pixel), compared to standard NMF for phase identification and to PCA for data set denoising. The PSNMF uses *b* = 15. Abundance maps and spectra from NMF and PSNMF are compared to ground truths. On the bottom, raw, PCA-denoised and PSNMF-denoised Nd maps are compared to the ground truth map.

Having quantitatively tested PSNMF on simulated data sets, we now validate its effectiveness on experimental data. The first sample studied is equivalent to that represented by the simulated data sets, having a nanometric distribution of Fp and CaPv phases in a Brg matrix. The sample was prepared using focused ion beam milling from a mineral assemblage synthesized under the conditions found in literature.^29^ The acquired data set has a spatial size of 512 × 512 pixels and an average of 120 X-ray counts per pixel, therefore closely resembling the counting statistics of the medium SNR synthetic data set. Figure 4 presents the results of PSNMF on this data set, again compared to standard NMF. Note that, while we cannot compare the component outputs to any ground truth maps, we are able to compare to ground truth spectra, which we have calculated from the original data set using a combination of NMF-derived masking and compositional prior knowledge, as described in our previous work.^34^ We conducted PSNMF experiments with binning factors *b* = 2, 4, 8, 16, 32; out of these, *b* = 8 gives the lowest average spectral angles, which are respectively just 0.15°, 0.96°, and 2.64° for Brg, Fp, and CaPv. Given that, unlike the method used to obtain the ground truth spectra,^34^ these high fidelity spectra are obtained from spatially and chemically overlapped phases without recourse to any prior knowledge about phase-distinguishing elements, this demonstrates the powerful capability of our approach. Figure 4 further demonstrates how our methodology identifies clear distribution maps of all three phases. In contrast, while standard NMF retrieves Brg and Fp distributions, with moderate spectral angles of 5.09° and 8.94°, it fails to retrieve the CaPv component, both spatially and spectrally (spectral angle = 71.29°). Turning to denoising, Figure 4 shows that the map of Nd from the PSNMF-reconstructed data set is very clear, even though it is a trace element in the Brg matrix (at a level of approximately 180 ppm). Further to this, we present spectra integrated from the same Brg-located 4 × 4 pixels in the raw, PCA-reconstructed and PSNMF-reconstructed data sets, to compare the *L*α peaks of trace rare earth elements Nd and Sm. The raw spectrum does not show any spectral shape, nor evidence of Nd and Sm. The PCA spectrum shows some spectral shape, and indicates some presence of Sm. However, clear Nd and Sm peaks are revealed using PSNMF. Moreover, this spectral signature matches well a Brg ground truth spectrum, which was itself determined by integrating 1.6 × 10^5^ pixel spectra, thereby vindicating the improved denoising of the PSNMF over the PCA processing.

**Figure 4:**
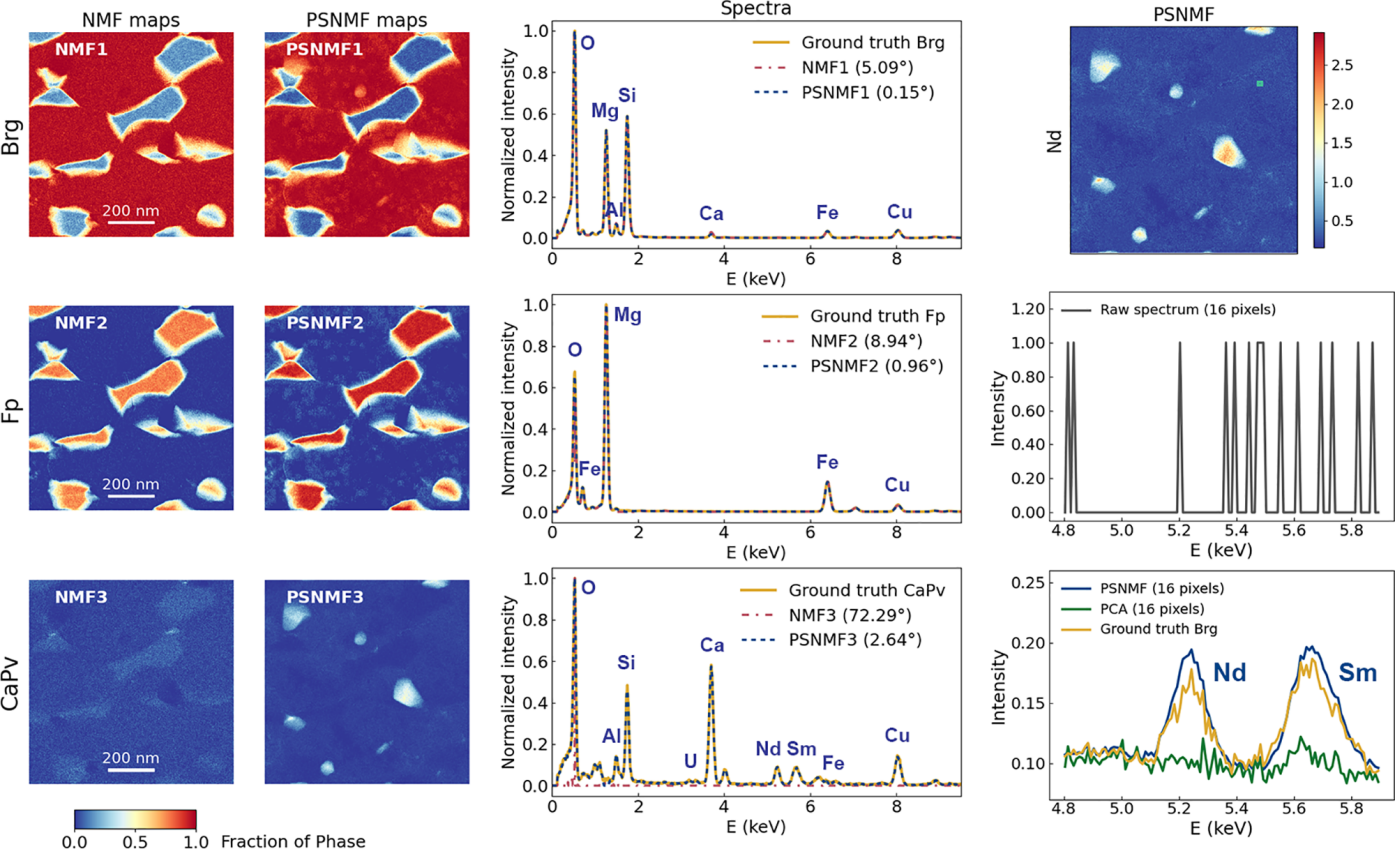
PSNMF applied to an experimental 512 × 512 pixel data set (having an average of 120 X-ray counts per pixel), acquired from a mineralogical sample. The PSNMF uses *b* = 8. The phase maps generated by PSNMF are compared to those determined using standard NMF. The phase spectra (middle column) are additionally compared to ground truth phase spectra calculated using the methodology described in Chen et al.^34^ In the right column, we study the denoising effect of the PSNMF-reconstructed data set, with the Nd map (top), and then EDX spectra integrated over the same 4 × 4 pixel Brg region (shown in green on the Nd map) from the raw data set (middle), and PCA- and PSNMF-reconstructed data sets (bottom). An appropriately scaled Brg ground truth spectrum is overlaid on the PCA/PSNMF spectra.

The mineralogical sample represents a major category of samples analyzed by STEM-EDX: lamellae of uniform thickness that are prepared from bulk materials using ion beam milling and/or mechanical thinning. We now test the effectiveness of PSNMF on the other major category of analyzed samples: nanostructures of irregular thickness that are supported on an amorphous carbon film. As a test object, we take a sample of Cu_2_O nanocubes of ∼20 nm size decorated with ∼3–4 nm diameter Au nanoparticles, which is being developed as an efficient catalyst for electrochemical CO_2_ reduction.^38^ Like many heterogeneous catalysts, characterizing and quantifying the size and distribution of the nanoparticles is of high scientific utility. However, as shown in Figure 5, while the larger Au nanoparticles are visible in the raw Au EDX map, their edges are indistinct, as for instance compared to the high angle annular dark-field STEM image shown in Supporting Information Figure S8. Further, many of the smallest Au nanoparticles cannot be distinguished at all. Both of these problems derive from the low SNR inherent for such small particles, which have very limited volumes for EDX signal generation.

**Figure 5:**
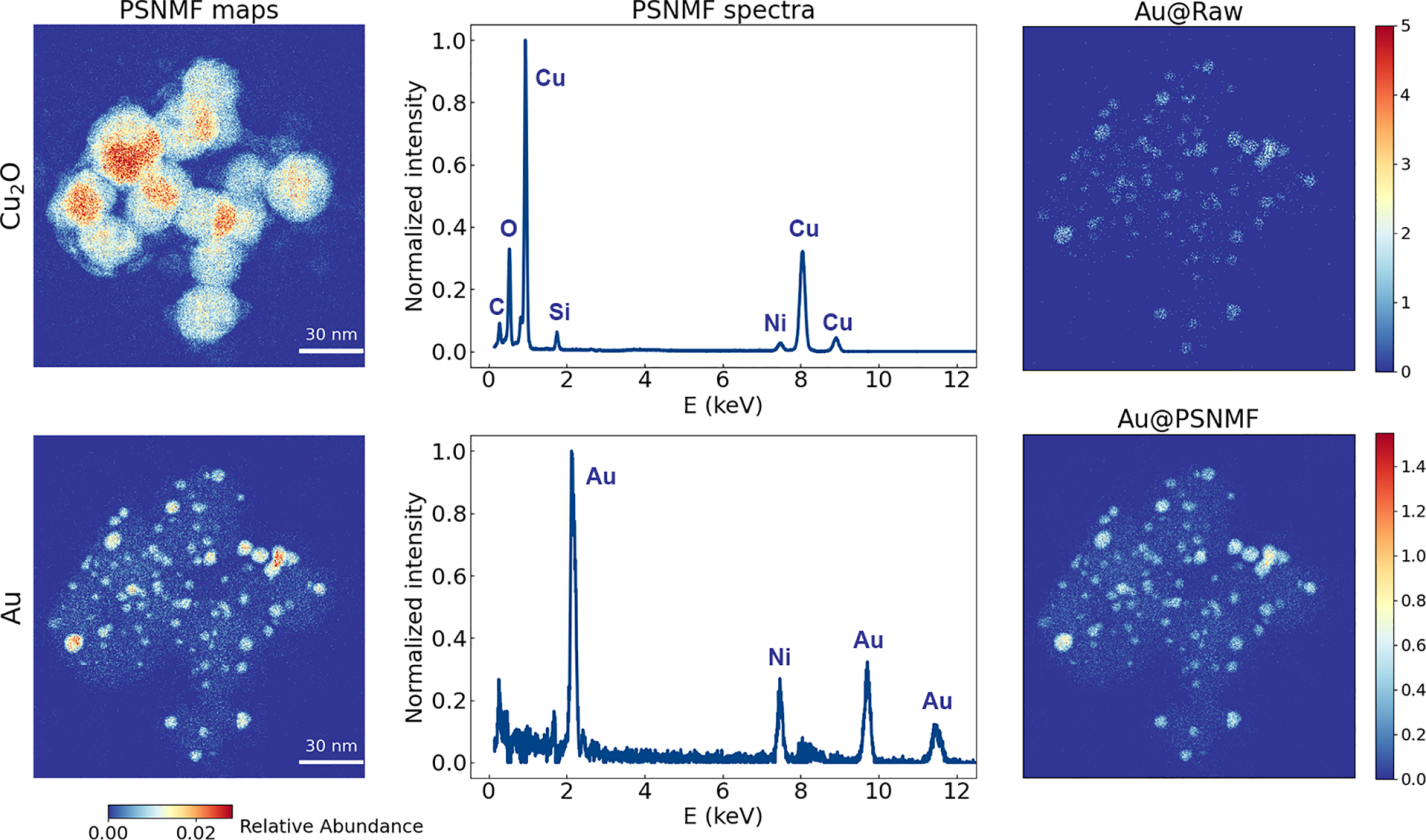
PSNMF applied to an experimental 512 × 512 pixel data set that was acquired from Au–Cu_2_O nanoparticles supported on a carbon film. The PSNMF uses *b* = 16. Two relevant phase maps and spectra generated by PSNMF are presented. In the right column, we examine the denoising effect of the PSNMF-reconstructed data set, as illustrated by the elemental Au map, in comparison to the raw data set.

Now applying PSNMF to the EDX data set with *b* = 16, Figure 5 shows that our method correctly separates the catalytic structures into their separate phases, producing low-noise abundance maps and component EDX spectra that correspond to the Cu_2_O nanocubes and the Au nanoparticles. From the Au abundance map alone, it is possible to quantify the sizes of the Au nanoparticles. As shown in Supporting Information Figure S9, two other phases are also identified, in turn corresponding to the amorphous carbon support film and a silica contamination. By reconstructing the EDX data set using PSNMF, the right-hand column of Figure 5 illustrates that we now achieve an elemental Au EDX map of low noise and high quality. Indeed, even Au nanoparticles of only 1.8 nm diameter are distinct from the background. This test case demonstrates that PSNMF is also an effective tool for the denoising and quantitative analysis of STEM-EDX data acquired from heterogeneous, supported nanostructures.

In conclusion, modern analytical transmission electron microscopes equipped with fast, single- or multisegment silicon drift EDX detectors have revolutionized nanoanalytics by giving researchers the capability to generate high-pixel-density elemental maps in a short acquisition time. However, quantitative interpretation of these results is commonly challenging, owing to a high level of spectral noise, together with EDX phase signals that are mixed together. We have presented PSNMF, a novel machine learning methodology for addressing both of these challenges, at the same time. By applying PSNMF to realistic synthetic data sets, we have proven its superior performance compared to benchmark algorithms. We have further illustrated its effectiveness on two experimental use-cases: first, a thinned lamella of a nanoscale multiphase system; second, heterogeneous nanoparticles supported on an amorphous carbon film. The high quality of phase decomposition and high fidelity of the denoised maps prove the strong potential of PSNMF for improving STEM-EDX analytics across a variety of nanoscience domains.

## Supporting Information Available

Mathematical description of PSNMF; raw elemental maps, PCA scree plots, and full comparisons of ground truth, raw, and PCA- and PSNMF-denoised elemental maps for medium and low SNR synthetic data sets; STEM image and PSNMF decomposition of experimental Au–Cu_2_O nanoparticle sample (PDF)
