# Supplementary material for: Leveraging Machine Learning for Advanced Nanoscale X-ray Analysis: Unmixing Multicomponent Signals and Enhancing Chemical Quantification
Source: Nano Lett. 2024 Aug 6;24(33):10177–85. doi: 10.1021/acs.nanolett.4c02446 (PMC11342375; doi:10.1021/acs.nanolett.4c02446)
Supplement: Supplementary file 1 — nl4c02446_si_001.pdf [file nl4c02446_si_001.pdf]

Supporting Information for "Leveraging Machine Learning for Advanced Nanoscale X-ray Analysis: Unmixing Multicomponent Signals and Enhancing Chemical Quantification"

Hui Chen, Duncan T.L. Alexander, and Cécile Hébert*

Electron Spectrometry and Microscopy Laboratory (LSME), Institute of Physics (IPHYS), École Polytechnique Fédérale de Lausanne (EPFL), 1015 Lausanne, Switzerland

E-mail: cecile.hebert@epfl.ch

Note 1. Mathematical description of PSNMF

We begin with the original noisy HR-LS (high-resolution, low-signal) dataset (Y) of size (y, e), where e is the number of energy channels (spectral features) and y is the number of pixels (spatial dimensions). Through spatial binning, we create the LR-HS (low-resolution, high-signal) dataset (X) of size (x, e), where x is the number of pixels and is calculated as $x = \frac{y}{b^2}$, with b being the bin size (e.g., a bin size of 4 results in $x = \frac{y}{16}$). We aim to combine the beneficial properties of Y and X into a new dataset Z. Z is a matrix of size (y,e) that possesses improved SNR (signal-to-noise ratio) while maintaining high spatial resolution.

The relationships between the datasets X, Y, and Z are described as follows:

$$X = ZS + E_s \tag{1}$$

$$Y = RZ + E_r \tag{2}$$

where S and R are transformation matrices, S is a spatial transformation matrix dictating the transformation of spatial features in Z for the low dimension dataset, and R is a spectral transformation matrix converting the spectral features in Z to match those in the lower spectral fidelity dataset. E_s and E_r are residuals. The data fusion problem is to estimate Z, which can be done via NMF (non-negative matrix factorization):

$$Z = WH + \epsilon \tag{3}$$

where W denotes spectral components, and H represents corresponding abundance maps; ϵ is the residual (error) that is assumed to be zero if W and H are accurately obtained.

The X and Y can be approximated similarly as follows:

$$X \approx WH_h \tag{4}$$

$$Y \approx W_m H \tag{5}$$

where H_h is the spatially reduced abundance matrix, and W_m is the spectrally deteriorated component matrix.

We first unmix the LR-HS dataset to retrieve an estimate for W and H_h; then we initialize the decomposition of the HR-LS dataset using W and upsampled H_h to obtain H. The two matrices W and H can be multiplied to obtain the fused dataset, Z. To implement this, we modified the standard version of the NMF algorithm,[1] by incorporating a "sum-to-one" constraint on the abundance matrix. This adaptation is specifically designed for use with

STEM-EDX data.

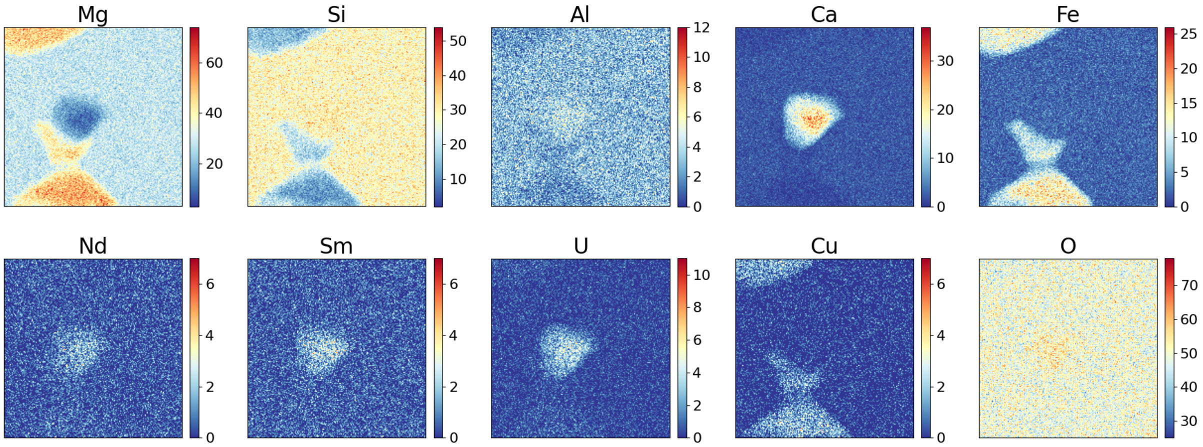

Figure S1: Raw elemental maps from the synthetic dataset with medium SNR.

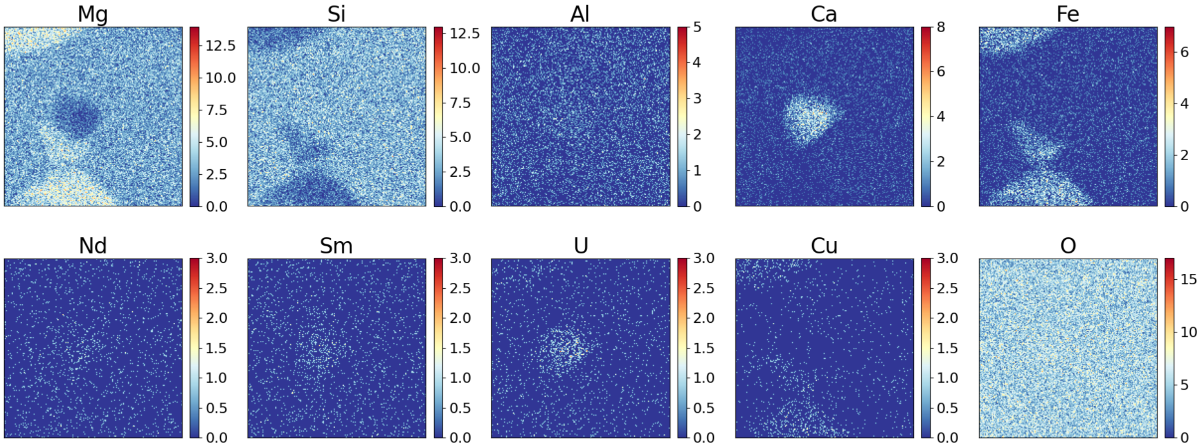

Figure S2: Raw elemental maps from the synthetic dataset with low SNR.

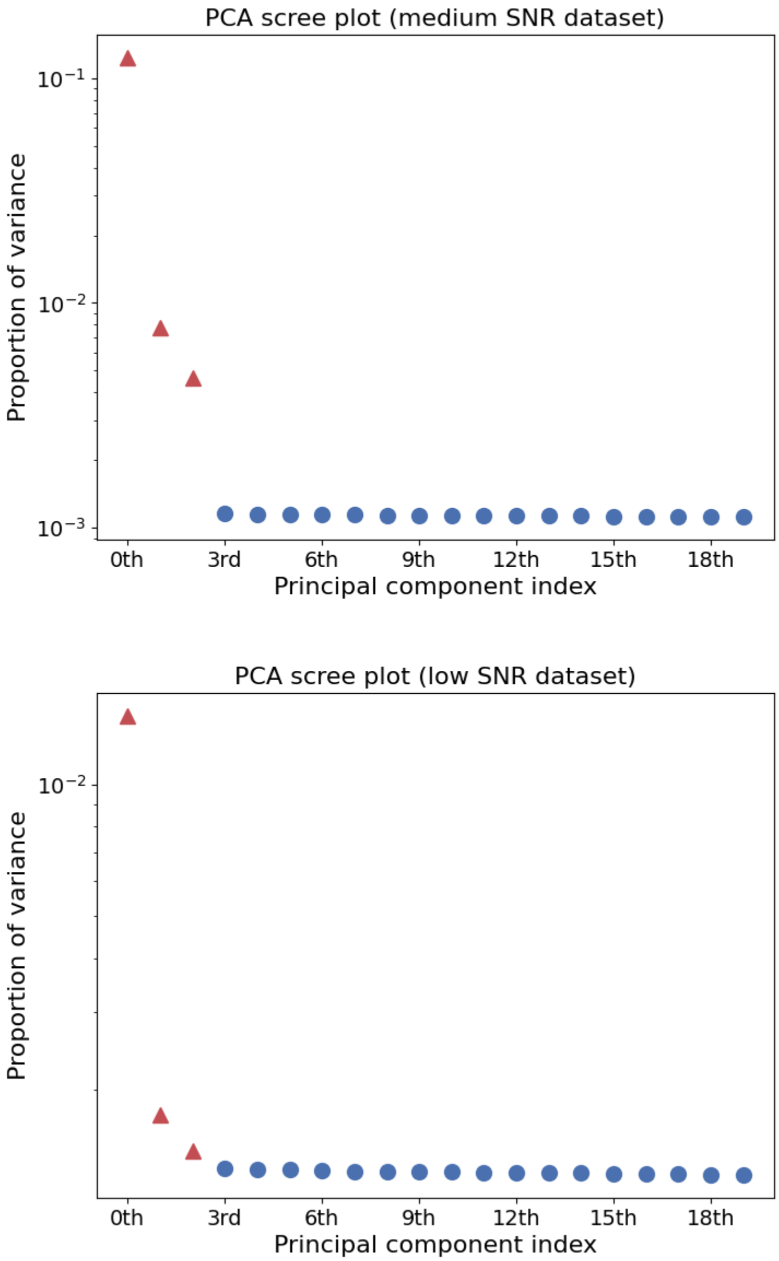

Figure S3: The scree plots of PCA decomposition of the medium SNR and low SNR dataset, respectively.

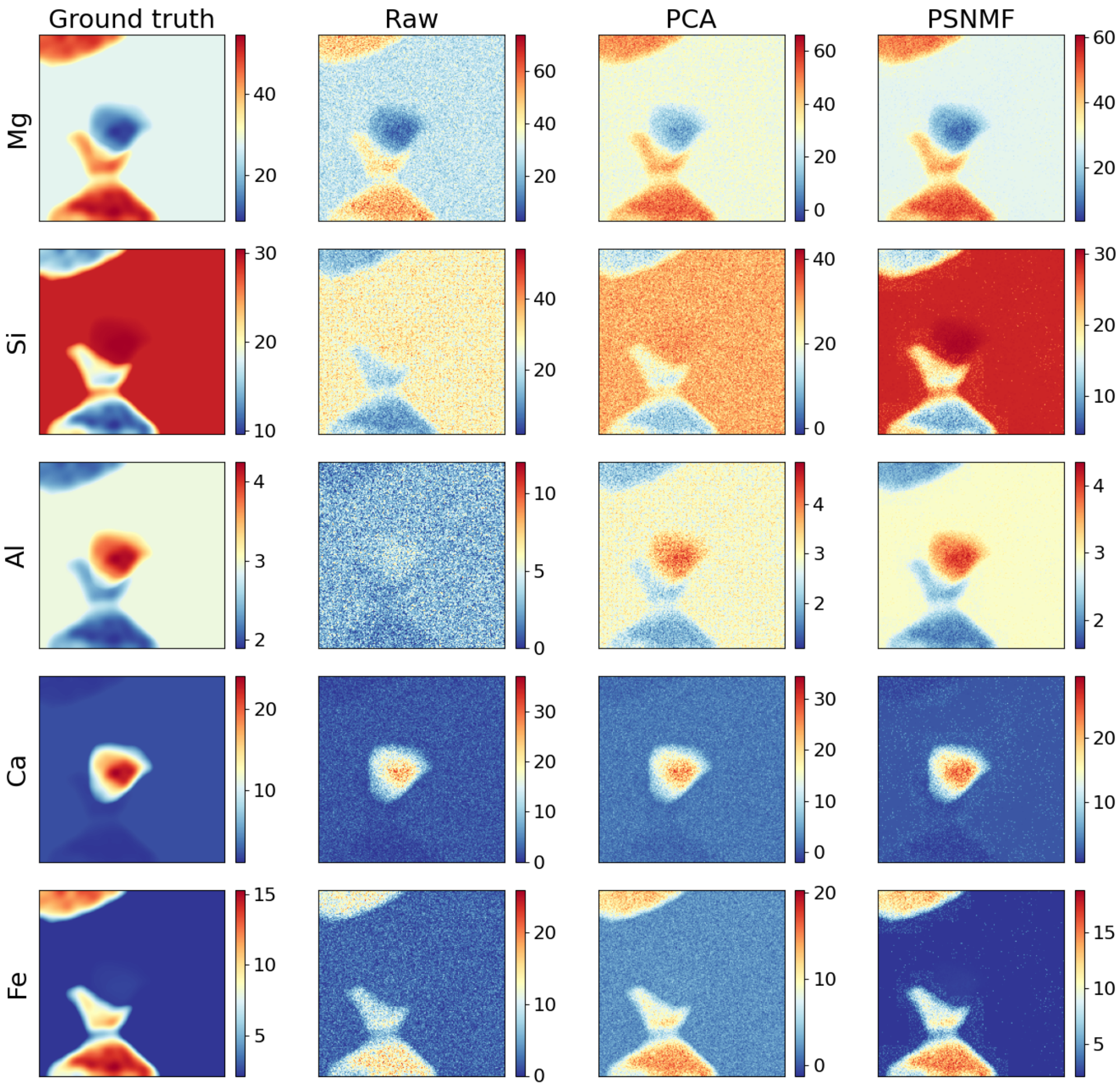

Figure S4: A full comparison of ground truth, raw, and PCA- and PSNMF-denoised elemental maps for medium SNR dataset (Part 1).

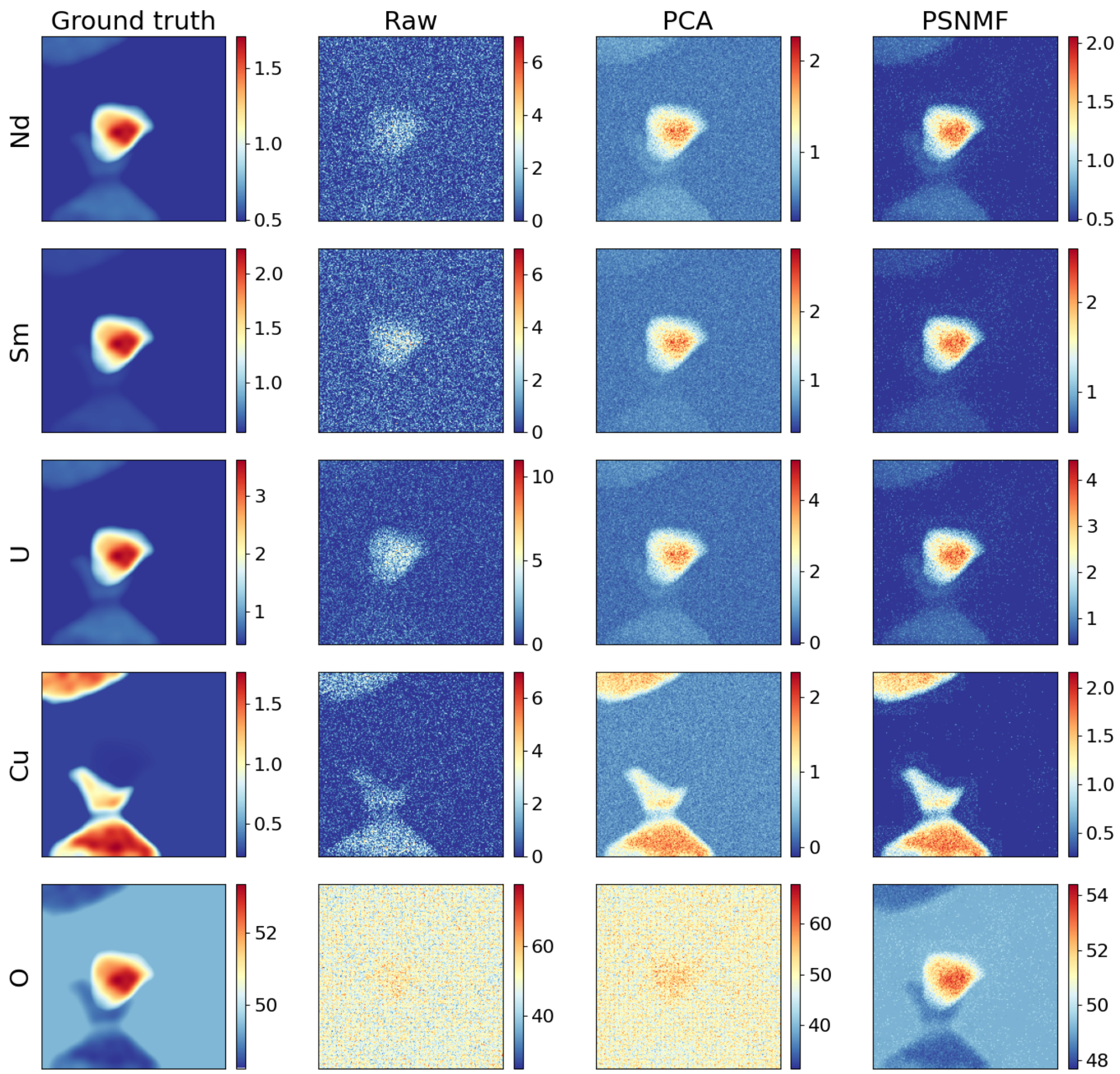

Figure S5: A full comparison of ground truth, raw, and PCA- and PSNMF-denoised elemental maps for medium SNR dataset (Part 2).

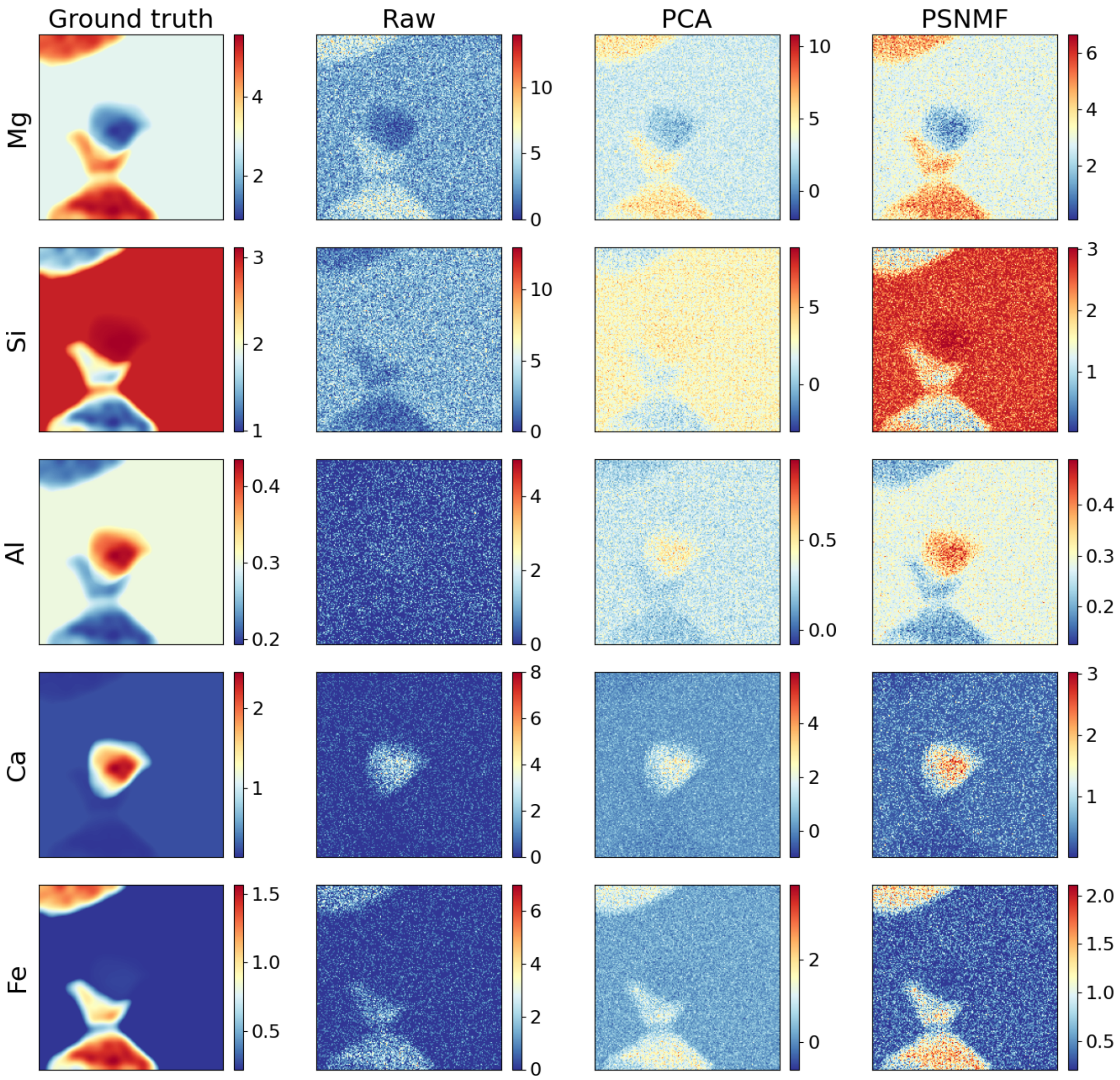

Figure S6: A full comparison of ground truth, raw, and PCA- and PSNMF-denoised elemental maps for low SNR dataset (Part 1).

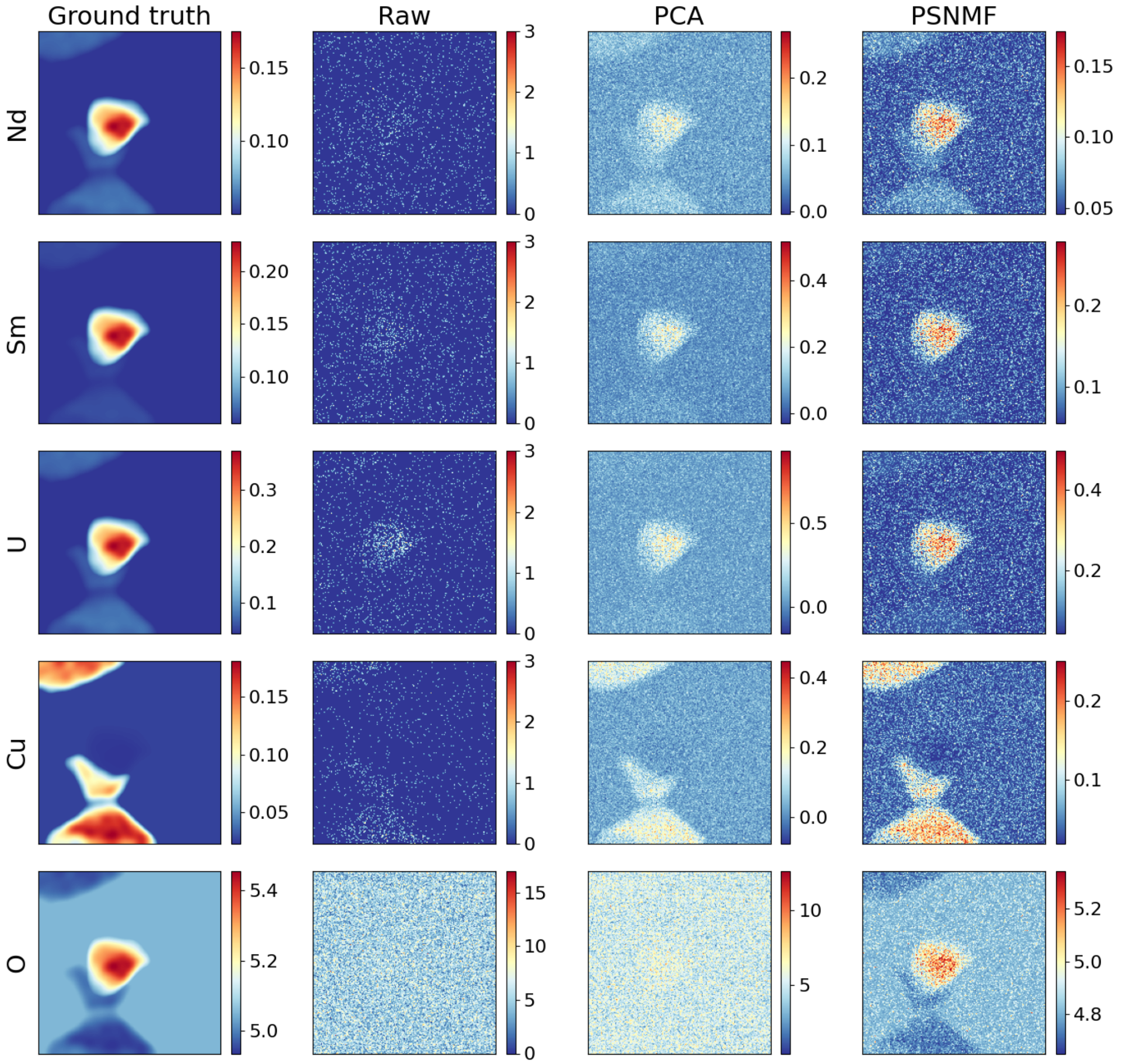

Figure S7: A full comparison of ground truth, raw, and PCA- and PSNMF-denoised elemental maps for low SNR dataset (Part 2).

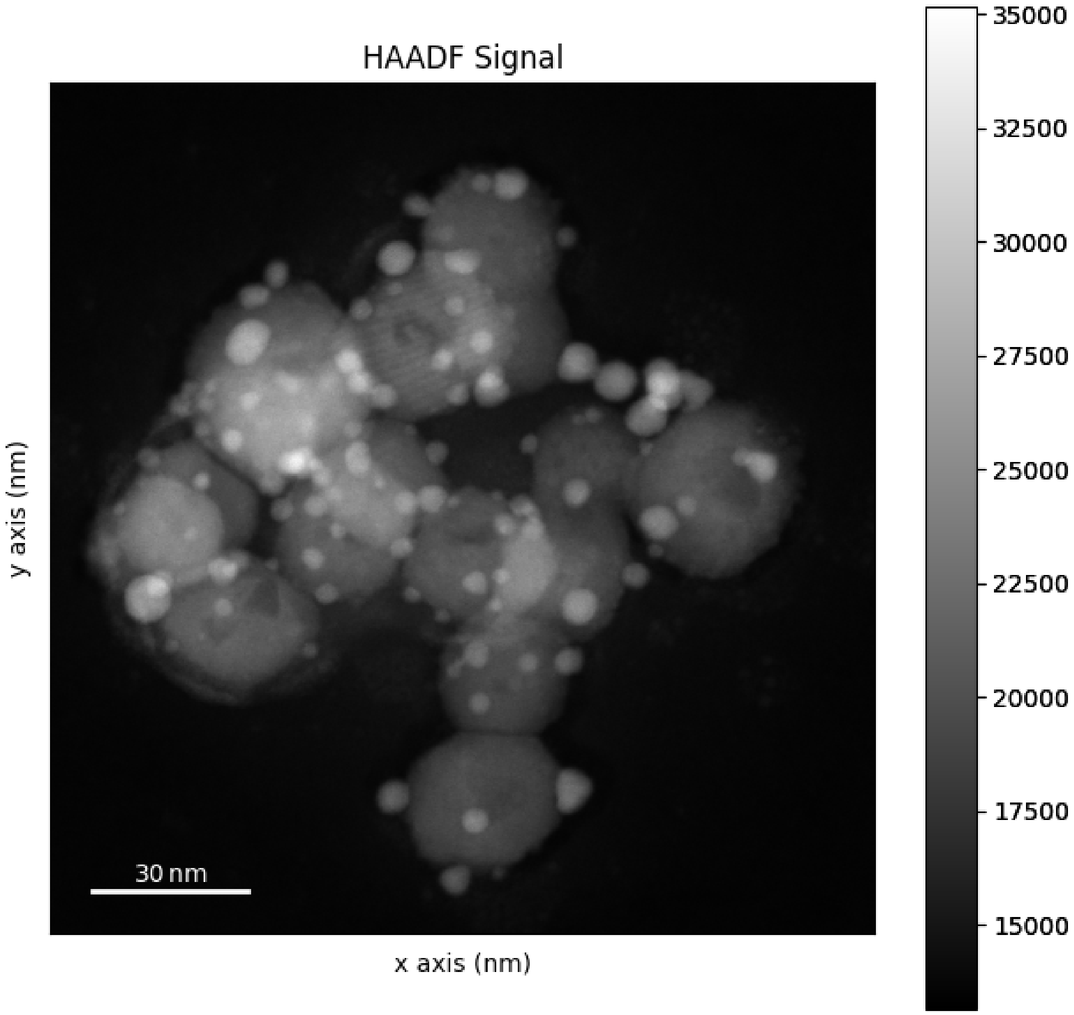

Figure S8: A high angle annular dark-field (Z-contrast) STEM image of the Au-Cu_2O nanoparticles supported on carbon.

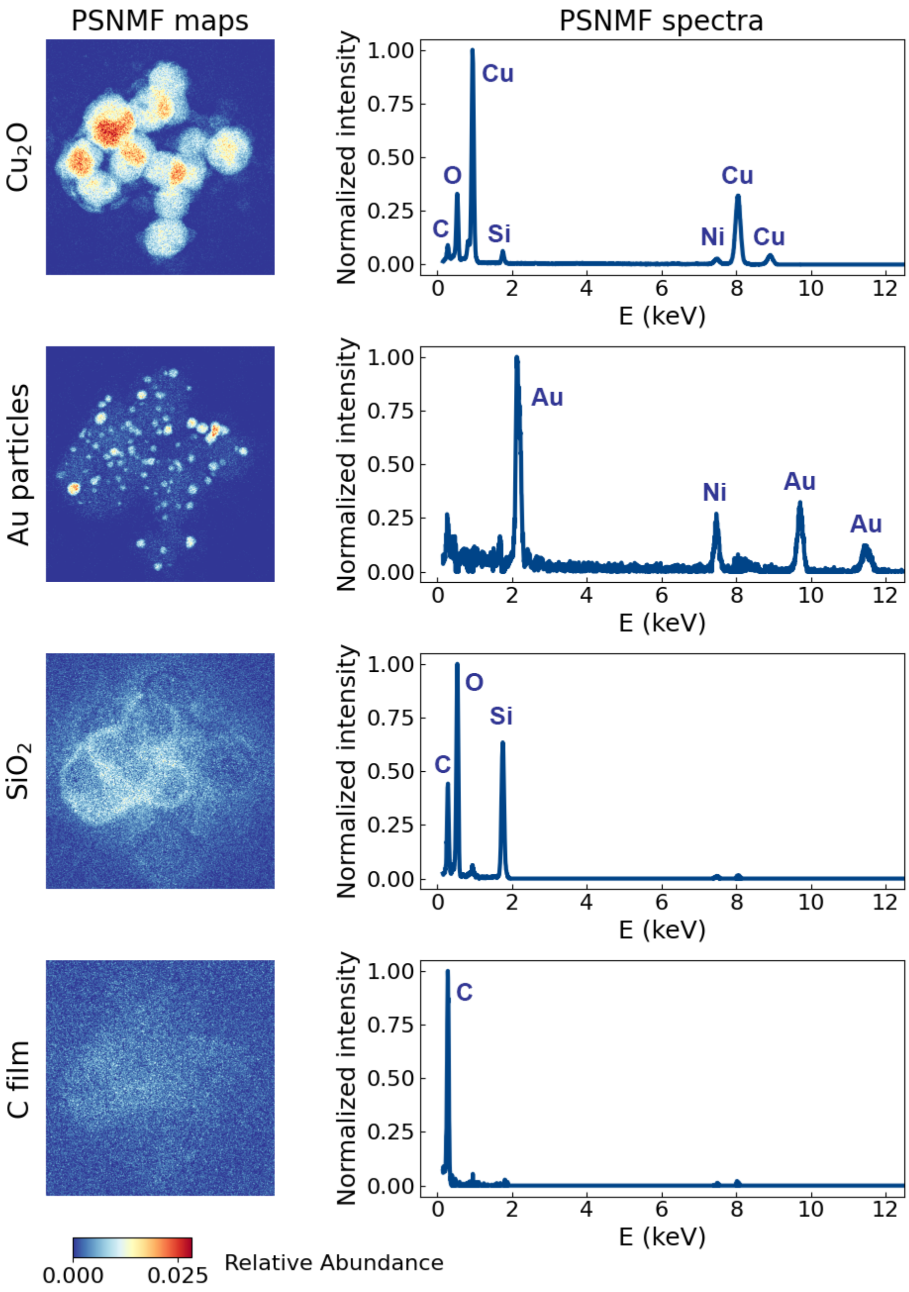

Figure S9: PSNMF decomposition results on analyzed nanoparticle sample.

References

(1) sklearn.decomposition.NMF. https://scikit-learn.org/stable/modules/generated/sklearn.decomposition.NMF.html, Accessed on 2023-12-7.
